# Interferon stimulation and NKG2D expression drive enhanced natural killer cell antibody-dependent cellular cytotoxicity against viral infections

**DOI:** 10.1093/jleuko/qiag019

**Published:** 2026-02-09

**Authors:** Leslie Chan, Kassandra Pinedo, Samuel Yang, Andra L. Blomkalns, Kari C. Nadeau, Angela J. Rogers, Catherine A. Blish

**Affiliations:** 1 Stanford Immunology Program, Stanford University School of Medicine, BioMedical Innovations Building, 240 Pasteur Drive, Stanford, CA 94305, United States; 2 Department of Medicine, Stanford University School of Medicine, 300 Pasteur Drive, Edwards Building Suite R106, Stanford, CA 94305, United States; 3 Department of Emergency Medicine, Stanford University School of Medicine, 900 Welch Road, Suite 350, Stanford, CA 94305, United States; 4 Department of Environmental Health, Harvard Chan School of Public Health and Department of Medicine, Harvard Medical School, 677 Huntington Avenue, Boston, MA 02115, United States; 5 Stanford Medical Scientist Training Program, Stanford University School of Medicine, 300 Pasteur Drive, Edwards Building Suite 133, Stanford, CA 94305, United States; 6 Chan Zuckerberg Biohub, 499 Illinois St, San Francisco, CA 94185, United States

**Keywords:** antibody-dependent cellular cytotoxicity, COVID-19, natural killer cells, NKG2D

## Abstract

Natural killer (NK) cell antibody-dependent cellular cytotoxicity (ADCC) contributes to effective antiviral immunity, yet the relative contribution of NK cell-intrinsic factors and antibodies in mediating these responses remains poorly understood. Here, we combined functional ADCC assays with single-cell transcriptomics of peripheral NK cells from COVID-19 participants. Our analysis revealed distinct transcriptional programs between participants with different ADCC response levels: NK cells from participants with lower ADCC responses upregulated proliferation pathways, while those with high ADCC responses showed enhanced expression of interferon-stimulated genes and NKG2D. Blocking NKG2D significantly reduced NK cell ADCC degranulation and cytokine responses. Paradoxically, greater interferon-mediated NK cell activation was associated with reduced proficiency of participants’ antibodies to mediate ADCC, suggesting a regulatory checkpoint mechanism. These findings enhance our understanding of the molecular determinants of ADCC responses and provide novel insights into leveraging these responses for more effective vaccination and therapeutic strategies.

## 1. Introduction

Antibodies provide critical protection against infections through multiple mechanisms. As novel viral variants emerge that evade neutralizing antibodies—a persistent challenge for vaccine development against viruses like the influenza virus, severe acute respiratory syndrome coronavirus 2 (SARS-CoV-2), and human immunodeficiency virus (HIV)—non-neutralizing antibody functions become increasingly important. Non-neutralizing antibodies mediate their protective functions by engaging innate immune cells, including natural killer (NK) cells, through a process called antibody-dependent cellular cytotoxicity (ADCC).^1–3^ ADCC responses have distinct and important roles in disease. ADCC activity correlates with favorable outcomes in SARS-CoV-2 and HIV-1 infections and protection against the influenza virus.^4–9^ Further, in HIV-1-exposed infants, antibodies capable of mediating ADCC are correlated more strongly with lower rates of mother-to-child transmission and lower postinfection infant mortality than neutralizing antibodies.^2^

While the role of non-neutralizing antibodies in ADCC activity is well-characterized, less attention has focused on NK cell determinants of effective ADCC responses. During ADCC, NK cells bind to antibody-coated target cells via FcγRIIIa (CD16) receptors, triggering the release of antiviral cytokines, such as interferon gamma (IFNγ) and tumor necrosis factor alpha (TNFɑ), as well as cytotoxic granules that directly eliminate infected target cells. Although CD16 polymorphisms are known to affect ADCC responses,^10^ other NK cell-intrinsic factors influencing ADCC efficacy remain largely unexplored. To address this gap, we combined ADCC functional assays with single-cell transcriptomics to perform a detailed assessment of the respective contributions of peripheral NK cells and plasma antibodies to ADCC responses in SARS-CoV-2-infected participants. This work identified the role of interferon-mediated activation and NKG2D expression in driving NK cell ADCC responses, offering new perspectives for enhancing NK cell ADCC activity against SARS-CoV-2.

## 2. Methods

### 2.1 Cell lines

Raji (#CCL-86) and K562 cells (#CCL-243) were purchased from ATCC. CEM.NKr.Spike cells, stably expressing the SARS-CoV-2 Spike D614G, were gifted by Dr. Andrés Finzi.^11^ Raji, CEM.NKr.Spike, and K562 cells were all cultured in complete RPMI-1640 medium (RPMI-1640 (Thermo Scientific #21870092) supplemented with 10% fetal bovine serum (FBS; Corning Ref #35–016-CV), 1× penicillin/streptomycin/amphotericin B (PSA; Gibco Cat #15240062 or Cytiva HyClone #SV30079.01), and 2 mM l-glutamine (Thermo Scientific #25030081)) at 37 °C, 5% CO_2_.

### 2.2 Clinical cohorts and samples

Cryopreserved peripheral blood mononuclear cells (PBMCs) were obtained from COVID-19 participants enrolled in the Stanford University COVID-19 Biobanking studies (Stanford IRB approvals #55650, 68301) from April to December 2021 (Table S1). Disease severity was scored using the WHO severity score (0 to 8), as previously described; all participants had a WHO score of 3 or 4.^12^ Participants categorized with a score of 3 had a mild to moderate disease, required hospitalization, but no supplemental oxygen. A score of 4 was given to hospitalized individuals requiring non-invasive supplemental oxygen.

### 2.3 NK cell isolation

After removal of 2.5 × 10^5^ PBMCs for scRNA-seq, all remaining PBMCs were plated in U-bottom 96-well plates (Thermo Scientific #163320) and rested overnight at 37 °C. The next day, NK cells were isolated from these PBMCs via negative selection, using the Miltenyi Biotec Human NK Cell Isolation Kit (#130-092-657), per manufacturer’s instructions.

### 2.4 NK cell ADCC functional assay

To evaluate NK ADCC functional responses, isolated NK cells were co-cultured with CellTrace Violet (Thermo Fisher Scientific #C34557)-labeled target cells with or without a monoclonal antibody at a 1:4 effector:target cell ratio for 6 h at 37 °C in complete RPMI-1640. In the negative control baseline condition, target cells were not coated with monoclonal antibodies. In the ADCC response condition, Raji target cells were coated with 1 μg/mL rituximab (Invivogen #hcd20-mab13), while CEM.NKr.Spike cells were coated with 10 μg/mL Fc-enhanced SARS-CoV-2 Spike monoclonal antibody mutant CV3–25 GASDALIE (gifted by Dr. Andrés Finzi).^13^ 1× Brefeldin A (eBioscience #00-4506-51), 1× monensin (eBioscience #00-4505-51), and anti-CD107a antibody (Table S2) were added at the beginning of the 6-h incubation. After the 6-h incubation, cells were stained with eBioscience Fixable Viability Dye eFluor 780 (eBioscience #65-0865-14) for 20 min at room temperature, followed by washing with phosphate-buffered saline (PBS; Thermo Scientific #10010049). Cells were then stained with a surface antibody panel for 30 min at room temperature (Table S2). After washing with FACS buffer (1× PBS, 2% FBS, 0.5% bovine serum albumin (BSA; Thermo Scientific #15260037)), cells were subsequently lysed with 1× BD FACS Lysing Solution (BD #349202) for 10 min and permeabilized with 1× BD FACS Permeabilizing Solution 2 (BD #340973) for 10 min at room temperature. This was followed by intracellular staining for 30 min at room temperature (Table S2), then washing with FACS buffer and fixation with 2% paraformaldehyde (PFA; EMS #15710). After fixation, samples were washed and resuspended in FACS buffer, then stored at 4 °C until data collection on an Aurora flow cytometer (within 3 d of staining).

Data analysis was performed using FlowJo version 10.8.1. NK cell responses were determined by subtracting baseline responses (without monoclonal antibody) from ADCC responses (with monoclonal antibody) for each participant sample.

### 2.5 NKG2D blocking

To evaluate the role of NKG2D in NK ADCC responses, isolated NK cells were first stimulated with 300 IU/mL IL-2 (R&D Systems #202-IL) overnight at 37 °C in complete RPMI medium. The next day, stimulated NK cells were treated with either 10 μg/mL monoclonal anti-human NKG2D antibody (BioLegend #320802) or 10 μg/mL monoclonal IgG1, κ isotype control antibody (BioLegend #401408) for 30 min at 37 °C. Immediately after, blocked NK cells were co-cultured with CellTrace Violet-labeled target cells with or without a monoclonal antibody as described above.

### 2.6 NK cell ADCC killing assay

To evaluate direct killing of target cells, NK cells were co-cultured with CellTrace Violet-labeled target cells with or without a monoclonal antibody, as described above, at a 4:1 effector:target cell ratio for 3 h. After the 3-h incubation, cells were stained with eBioscience Fixable Viability Dye eFluor 780 (eBioscience #65-0865-14) for 20 min at room temperature, followed by washing with PBS (Thermo Scientific #10010049). Cells were then stained with a surface antibody panel for 30 min at room temperature (Table S3) and fixed with 2% PFA (EMS #15710). After fixation, samples were washed and resuspended in FACS buffer, then stored at 4 °C until data collection on an Aurora flow cytometer (within 3 d of staining).

### 2.7 Plasma ADCC assay

PBMCs from a healthy donor were thawed and rested overnight at 37 °C in complete RPMI medium. The same batch of PBMCs from the same donor was used across all batches. The next day, these PBMCs were co-cultured with CellTrace Violet-labeled CEM.NKr.Spike cells in RPMI medium supplemented with penicillin-streptomycin only (no FBS) at a 10:1 effector:target cell ratio with and without 1:500 participants’ plasma for 4 h at 37 °C. Brefeldin A, monensin, and anti-CD107a antibody were added at the beginning of the 4-h incubation. After the 4-h incubation, cells were stained, acquired, and analyzed as above except with a different antibody panel (Table S4).

### 2.8 Single-cell RNA sequencing

Concurrently with PBMC processing for NK cell ADCC assays, 2.5 × 10^5^ cells from each sample were fixed and processed for single-cell RNA sequencing using the Evercode Cell Fixation v2 (#ECF2001) and WT Mega v2 (#ECW02050) kits from Parse Biosciences according to the manufacturer’s instructions. Immediately after fixation, cells were placed into a Mr. Frosty and stored at −80 °C. After all samples were fixed, samples underwent 3 rounds of split-pool barcoding in a 96-well plate. The cells were subsequently lysed, and the released barcoded cDNA was amplified, fragmented, and size selected for sequencing. cDNA quality and expected peak between 400 and 500 base pairs were confirmed by TapeStation prior to sequencing. Sequencing was performed on a NovaSeq S4 instrument (Illumina; Chan Zuckerberg Biohub).

### 2.9 Statistical analysis

Participants were stratified into low and high ADCC response groups using the mean ADCC degranulation response as the cutoff. This provided more balanced group sizes compared to median-based stratification (which differed by only 1 participant). Differences between groups were assessed by the Wilcoxon Rank-Sum test with Benjamini–Hochberg’s correction for multiple hypothesis testing. *P ≤ 0.05, **P ≤ 0.01, ***P ≤ 0.001, ****P ≤ 0.0001. All data analysis was performed using R versions 4.2.0 and 4.2.2.

## 3. Results

### 3.1 Proportions of CD56^dim^ and proliferating NK cells correlate with ADCC response variability

**Fig. 1.**
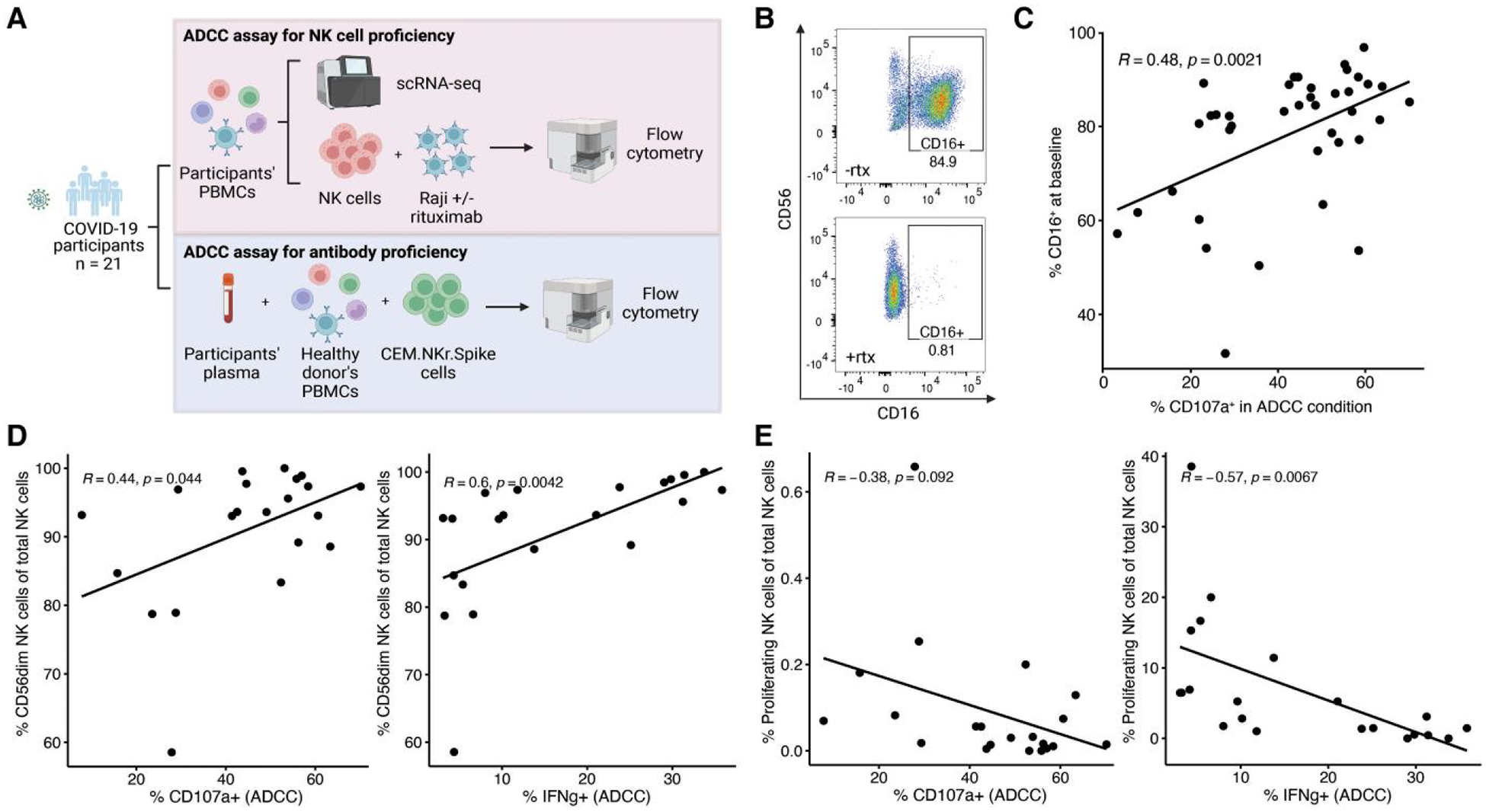
NK cell ADCC responses in COVID-19 participants. A) Pipeline of PBMC and plasma sample (*n* = 21) processing for paired scRNA-seq and ADCC functional assays. B) Representative flow cytometry plots of CD3^−^CD14^−^ NK cells when co-cultured with Raji cells in the absence and presence of rituximab (rtx). C) Scatterplot depicting the correlation between NK cell ADCC degranulation (CD107a) response and baseline CD16 expression. D and E) Scatterplot depicting the correlation between NK cell ADCC CD107a and IFNγ responses and the proportion of CD56^dim^ NK cells D) and proliferating NK cells E) out of total NK cells.

To identify factors shaping the NK cell contribution to ADCC responses in COVID-19 participants, we performed paired single-cell RNA sequencing (scRNA-seq) of PBMCs and ADCC functional assays using isolated NK cells from SARS-CoV-2-infected participants co-cultured with rituximab-coated Raji cells (Fig. 1A and Table S1). NK cell ADCC responses were assessed by their expression of the degranulation marker CD107a and the cytokine IFNγ (Fig. S1A and S1B). Consistent with prior work, we identified that expression of the NK cell ADCC receptor CD16 correlated positively with ADCC degranulation responses (Fig. 1B and 1C).^10^

In the scRNA-seq dataset, we next examined the proportions of CD56^dim^, CD56^bright^, and proliferating NK cells identified by mapping to an annotated multimodal PBMC reference (Fig. S2A–S2D).^14^ CD56^bright^ NK cells were marked by upregulated *NCAM1* expression, proliferating NK cells by upregulated *MKI67* expression, and CD56^dim^ NK cells by downregulated *NCAM1* expression and upregulated *PRF1* expression (Fig. S2A). We observed that ADCC degranulation (CD107a) and IFNγ responses showed a positive correlation with the proportion of CD56^dim^ NK cells (CD107a: R = 0.44, *P* = 0.044; IFNγ: R = 0.6, *P* = 0.0042) and a negative correlation with the proportion of proliferating NK cells (CD107a: R = −0.38, *P* = 0.092; IFNγ: R = −0.57, *P* = 0.0067; Fig. 1D and 1E).

### 3.2 Interferon-stimulated gene expression and NKG2D drive enhanced ADCC responses

**Fig. 2.**
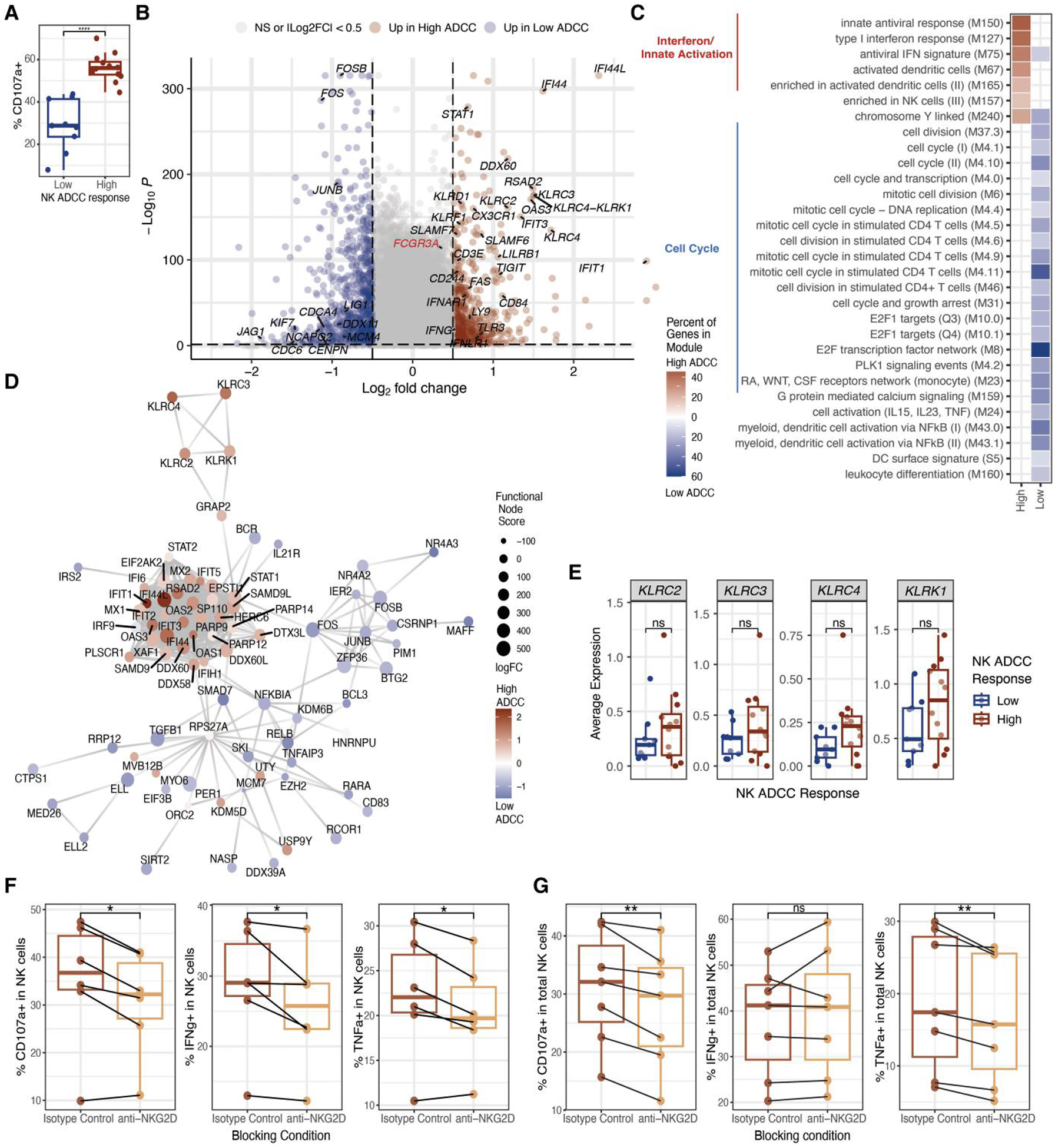
Interferon-mediated NK cell activation and NKG2D activity are associated with greater NK cell ADCC responses. A) NK cell ADCC degranulation activity is measured as the percent of NK cells staining positive for CD107a when co-cultured with rituximab-coated Raji cells at a 1:4 NK cell:Raji cell ratio for 6 h, subtracted from baseline activity when co-cultured with uncoated Raji cells. ADCC responses are stratified as low (*n* = 9) or high (*n* = 12) if they are below or above the mean ADCC degranulation response, respectively. B) Volcano plot depicting differentially expressed genes (DEGs) upregulated in NK cells from participants with low vs high ADCC responses. C) Heatmap displaying blood transcriptional modules (BTMs) enriched in DEGs identified in B). The percent of genes in the module was calculated by determining the number of genes enriched in each group out of the total number of measured module genes. D) The top 500 DEGs (ranked by the absolute value of log-fold change) identified in B) were mapped to known protein–protein networks derived from the human STRING database. Each gene is assigned a score derived from a β-uniform mixture model fitted to the *P*-values generated from DEG analysis. The highest scoring subgraph was generated with log-fold change relative to the high ADCC response group, as indicated by the color scale. E) Box plots depicting the mean expression of genes encoding for NK receptors in NK cells. F and G) Boxplots depicting CD107a, IFNγ, and TNFɑ expression in NK cells co-cultured with rituximab-coated Raji cells F) or SARS-CoV-2 Spike monoclonal antibody-coated CEM.NKr.Spike cells G) at an effector: target cell ratio of 1:4. Expression levels are subtracted from baseline expression in NK cells co-cultured with target cells without antibody coating. ns, not significant; *, *P* ≤ 0.05; **, *P* ≤ 0.01; ***, *P* ≤ 0.001 by Wilcoxon Rank-Sum test with Benjamini–Hochberg’s correction for multiple hypothesis testing.

To identify transcriptomic predictors of ADCC capacity in NK cells, we stratified participants’ NK cell ADCC responses as low or high ADCC response groups based on whether their degranulation responses were below or above the mean response level (44.54%) (Fig. 2A). We next performed differential gene expression analysis comparing NK cell transcriptomics in the 2 groups (Fig. 2B). NK cells from participants with high ADCC responses upregulated interferon stimulated genes (ISGs) and receptors and signaling molecules upstream of ISG induction (Fig. 2B–2D). While prior work showed reduced ISG induction in NK cells from SARS-CoV-2-infected participants with prior vaccination,^15^ vaccination status did not affect participants’ NK cell ADCC response (Fig. S3A and S3B).

In addition to ISGs, NK cells from participants with high ADCC responses upregulated several NK receptor and signaling genes, including *KLRC2* (NKG2C), *KLRK1* (NKG2D), *KLRC3* (NKG2E), and *KLRC4* (NKG2F) (Fig. 2B and 2D). Genes encoding these NK cell receptors trended toward higher expression in participants with high ADCC responses (Fig. 2E). The upregulation of *KLRC2* (encoding NKG2C) aligns with greater ADCC responses in adaptive-like NKG2C ^+^ CD57^+^ NK cells (Fig. S3C and S3D) as previously reported.^16,17^ Confirming the role of NKG2D, blocking NKG2D significantly reduced NK cell ADCC degranulation and cytokine responses against rituximab-coated Raji cells and SARS-CoV-2 Spike monoclonal antibody-coated CEM.NKr.Spike cells (Figs. 2F and 2G, and S3E). This was not recapitulated when ADCC killing of target cells was directly evaluated, demonstrating that NKG2D activity alone is not sufficient to consistently drive ADCC killing (Fig. S3F and S3G). The lack of NKG2E- and NKG2F-specific antibodies precluded in vitro confirmation of their role in mediating NK cell ADCC responses. Consistent with the positive correlation between CD16 protein expression and NK cell ADCC responses (Fig. 1C), we found that *FCGR3A* (encoding CD16) is also upregulated in the high ADCC group (log2FC: 0.364, Fig. 2B).

In contrast, NK cells from participants with low ADCC responses upregulated cell cycle genes and Notch and Fos/Jun signaling genes (Fig. 2B–2D). These findings demonstrate transcriptional divergence toward either proliferation or antiviral profiles in NK cells from participants with low vs high ADCC responses, respectively.

### 3.3 Interferon-activated NK cells correlate with limited antibody proficiency to mediate ADCC

After investigating the contribution of participants’ NK cells to ADCC, we next turned to how participants’ plasma antibodies contribute to this response. To isolate antibody-specific contributions, we used plasma from COVID-19 participants as the antibody source while maintaining a constant NK cell source from a healthy donor to assess ADCC activity against CEM.NKr.Spike cells (Fig. 1A).^11^ Prior vaccination status did not affect participants’ antibody ADCC responses, nor did we observe any correlation between antibody ADCC capacity and the proficiency of participants’ NK cells to execute ADCC (Fig. S4A and S4B).

**Fig. 3.**
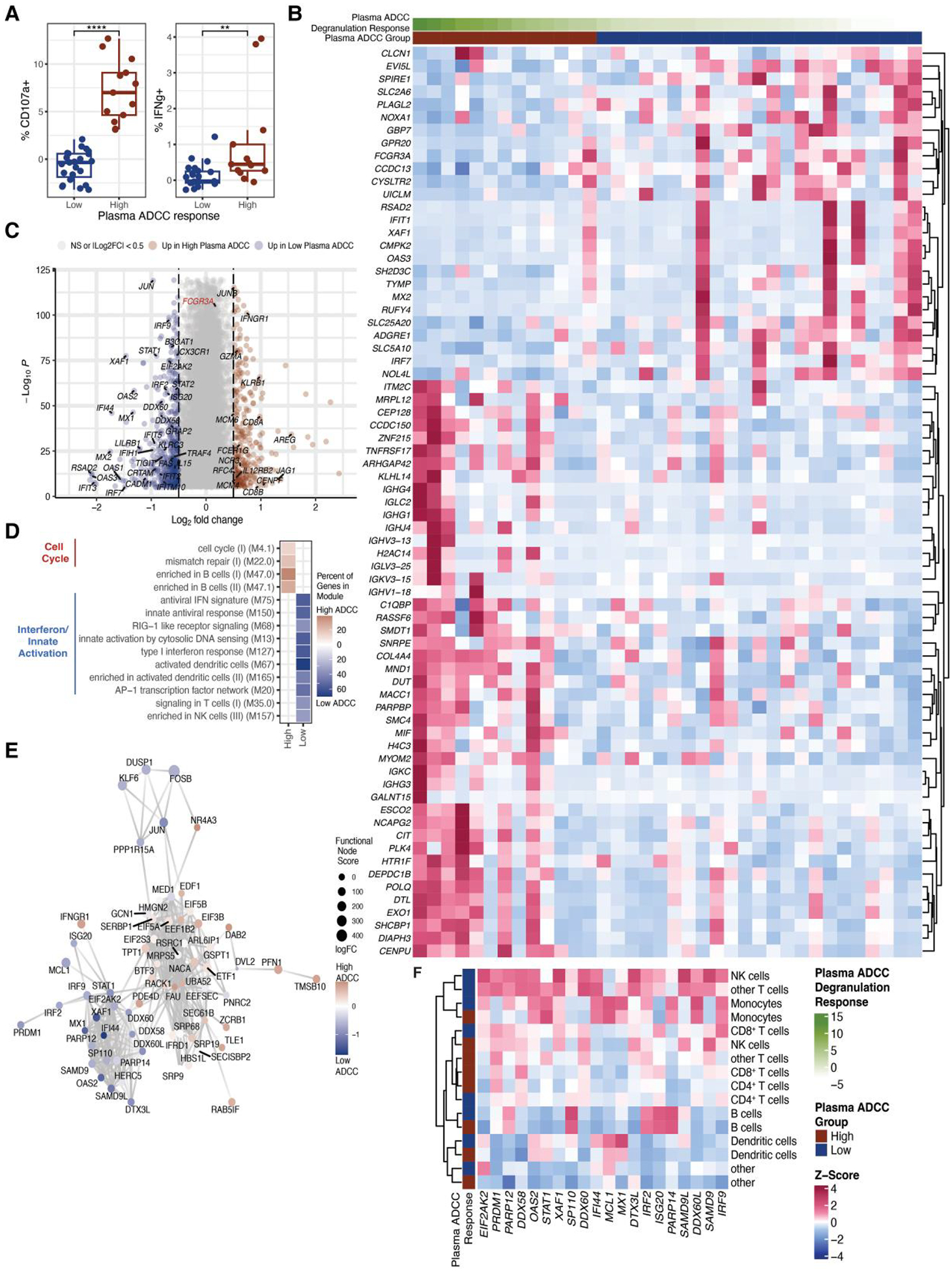
Interferon-mediated NK cell activation is associated with reduced antibody capacity for ADCC. A) Boxplots depicting the percent of healthy donor PBMCs staining positive for CD107a and IFNγ when co-cultured with CEM.NKr.Spike cells in the presence of participants’ plasma at 1:250 dilution at a 10:1 PBMC:CEM.NKr.Spike cell ratio for 4 h. ADCC responses are stratified as low (*n* = 23) or high (*n* = 13) if they are below or above the mean ADCC degranulation response, respectively. B) Heatmap displaying pseudobulk expression of genes significantly correlated with plasma-mediated ADCC responses in bulk PBMCs. C) Volcano plot depicting DEGs upregulated in NK cells from participants with antibodies that mediated low vs high ADCC responses. D) Heatmap displaying BTMs enriched in DEGs identified in C) as described in Fig. 2. E) The top 500 DEGs identified in C) were mapped to known protein–protein networks derived from the human STRING database as described in Fig. 2. F) Heatmap displaying pseudobulk expression of ISGs identified in E) in peripheral immune cell types from participants with antibodies that mediated low vs high ADCC responses.

To identify transcriptomic correlates of plasma-mediated ADCC responses, we stratified participants’ responses as low or high based on whether degranulation responses fell below or above the mean response (1.23%) (Fig. 3A). Pseudobulk DESeq2 analysis^18^ revealed that PBMCs from participants with high antibody proficiency for ADCC upregulated immunoglobulin (*IG–*) genes and cell cycle-related genes (Fig. 3B). In contrast, PBMCs from participants with low antibody proficiency for ADCC upregulated ISGs and *FCGR3A* (Fig. 3B).

We postulated that the inverse relationship between ISG expression and antibody proficiency for ADCC responses could be linked to recent findings suggesting that interferon activation of NK cells contributes to suppressed antibody responses.^19^ We therefore compared the transcriptional profile of NK cells from participants with low vs high plasma-mediated ADCC responses (Fig. 3C). This analysis revealed that NK cells from participants with low plasma-mediated ADCC responses upregulated ISGs (Fig. 3C–3E). In contrast, NK cells from participants with high plasma-mediated ADCC responses upregulated genes involved in normal processes, including transcription, translation, cell cycle, and mismatch repair pathways, as well as *FCGR3A* modestly (Fig. 3D and 3E).

To determine whether the correlation between interferon-mediated activation and antibody-mediated ADCC responses was NK cell-specific, we compared pseudobulk expression of the ISG hub upregulated in NK cells (Fig. 3E) across multiple immune cell types: CD4^+^ T cells, CD8^+^ T cells, B cells, NK cells, monocytes, dendritic cells, and other T cells (Fig. 3F). The most pronounced induction of these ISGs occurred in NK cells and other T cells from participants with low plasma-mediated ADCC responses (Fig. 3F). These findings highlight the complex relationship between interferon-mediated NK cell activation and ADCC responses characterized by competing roles of NK cells as both direct effectors and as potential modulators of antibody-dependent functions.

## 4. Discussion

While NK cell ADCC responses contribute to effective immunity against cancer and infections,^6,8,20–23^ the factors mediating these responses remain incompletely understood. We therefore assessed the respective contribution of NK cell effectors and plasma antibodies in ADCC responses in the setting of SARS-CoV-2 infection. We found that lower NK cell ADCC potential in participants was correlated with a higher proportion of proliferating NK cells and upregulated expression of cell cycle genes, consistent with prior findings demonstrating a negative correlation between CD8^+^ T cell proliferation and cytotoxicity capacity.^24^ The upregulation of ISGs in NK cells with high ADCC responses aligns with previous findings that type I interferon priming enhanced NK cell ADCC against HIV-1-infected cells and influenza virus-infected cells.^25,26^ However, while this interferon-mediated activation enhances NK cell ADCC capacity, it also correlates with diminished antibody capacity for ADCC. This opposing relationship suggests an immunoregulatory checkpoint that restrains excessive NK cell ADCC responses during inflammation. This immunoregulatory model is consistent with recent work demonstrating that interferon-mediated NK cell activation is associated with reduced antibody neutralization breadth during SARS-CoV-2 infection.^19^ However, future studies are needed to better understand the impact of interferon-mediated NK cell activation on the quality of antibodies’ contribution to ADCC responses.

In addition to ISGs, we found that several NK receptors, including *KLRK1* encoding the NK activating receptor NKG2D, were upregulated in NK cells with high ADCC responses. NKG2D has been shown to synergize with CD16 to enhance antibody-dependent responses^27^ and has been implicated in NK cell ADCC responses during HIV-1 infection.^28,29^ Here, we confirm that NKG2D significantly enhances NK cell ADCC degranulation and cytokine responses, providing a potential target for therapeutic intervention. Because NKG2D contribution alone was insufficient to enhance direct ADCC killing, this raises a key limitation of this study that we did not measure direct ADCC killing in addition to degranulation and IFNγ responses from COVID-19 participants due to limited NK cells available per sample. Future studies should functionally validate additional transcriptomic correlates of ADCC responses given that our findings demonstrate that multiple mechanisms contribute to NK cell ADCC and further explore the regulatory mechanisms balancing NK cell activation and antibody-mediated ADCC.

In conclusion, functional and transcriptional profiling of NK cell ADCC responses during SARS-CoV-2 infection identifies interferon activation and NKG2D expression as key determinants of enhanced ADCC capacity. These findings provide valuable insights for engineering NK cells with more robust ADCC responses for treating cancer and viral infections.

## Supplementary Material

Supplementary material

Supplementary material is available at Journal of Leukocyte Biology online.

## Data Availability

Flow cytometry FCS files with de-identified metadata supporting this publication are available on Flow Repository under Repository IDs FR-FCM-Z77X and FR-FCM-Z77P and CytoBank. Data from scRNA-seq are deposited with the Gene Expression Omnibus under accession no. GSE261862. All original code used for analysis and visualization is available on Zenodo (DOI: 10.5281/zenodo.13972674 and 10.5281/zenodo.18273759).
