# Supplementary material for: Interferon stimulation and NKG2D expression drive enhanced natural killer cell antibody-dependent cellular cytotoxicity against viral infections
Source: J Leukoc Biol. Author manuscript; Available in PMC 2026 Apr 29. (PMC13127262; doi:10.1093/jleuko/qiag019)
Supplement: Supplementary material [file NIHMS2160418-supplement-Supplementary_material.docx]

**Supplementary Materials and Methods**

**Alignment and quality control of single-cell RNA sequencing data**

The Parse Bioscience processing pipeline (v1.0.3p) was used with default settings to align sequencing reads to the GRCh38 human genome modified to include the SARS-CoV-2 viral genome and to demultiplex samples. To remove putative multiplets, cells that had >7,500 unique genes or >20,000 total number of RNA molecules were filtered. Cells with >15% of reads from mitochondrial genes were considered to be of low quality and removed from further analysis.

**Pre-processing of single-cell RNA sequencing data**

The R package Seurat was used for data scaling, transformation, clustering, dimensionality reduction, differential expression analysis, and visualization[^14^](https://www.zotero.org/google-docs/?OrUXK5). The SCTransform() function was used to scale and transform data and identify variable genes[^31^](https://www.zotero.org/google-docs/?u6Y3RX). Linear regression was performed to remove unwanted variation due to cell quality (e.g., percentage of mitochondrial reads, percentage of rRNA reads). Principal component (PC) analysis (PCA) was performed for dimensionality reduction, and the first 50 PCs were used to perform UMAP to embed the dataset into 2 dimensions. Next, the first 50 PCs were used to construct a shared nearest neighbor graph (SNN; FindNeighbors()), and this SNN was used to cluster the dataset (FindClusters()).

For cell type annotation, the transcriptome dataset was mapped to an annotated multimodal reference[^14^](https://www.zotero.org/google-docs/?WoufsX). First, anchors between the query and reference dataset were identified using a precomputed supervised PCA on the reference dataset that maximally captures the structure of the weighted nearest neighbor graph. Next, cell type labels from the reference dataset were transferred to each cell of the query through the previously identified anchors. Finally, the query dataset was projected onto the UMAP structure of the reference.

**Differentially expressed genes analysis**

Genes with low expression and low variance were first filtered out by keeping only the genes in the top quartile in mean expression, followed by removing the genes in the bottom quartile in variance. Differentially expressed genes (DEGs) were then identified using Seurat’s FindMarkers() function, specifying MAST (Model-based Analysis of Single-cell Transcriptomics) as the method of choice and adding sample as a latent variable[^32^](https://www.zotero.org/google-docs/?0MmkHY).

DEGs were ranked by their absolute average log-fold change, and the top 500 DEGs with an absolute average log-fold change ≥ 0.25 were selected for network analysis. The list of DEGs used to seed the skeleton network was first filtered to remove any DEGs with an adjusted p-value > 0.05. These DEGs were then mapped to known protein-protein networks derived from the Search Tool for the Retrieval of Interacting Genes/Proteins (STRING) database using the BioNet R package[^33^](https://www.zotero.org/google-docs/?1YlPH5).


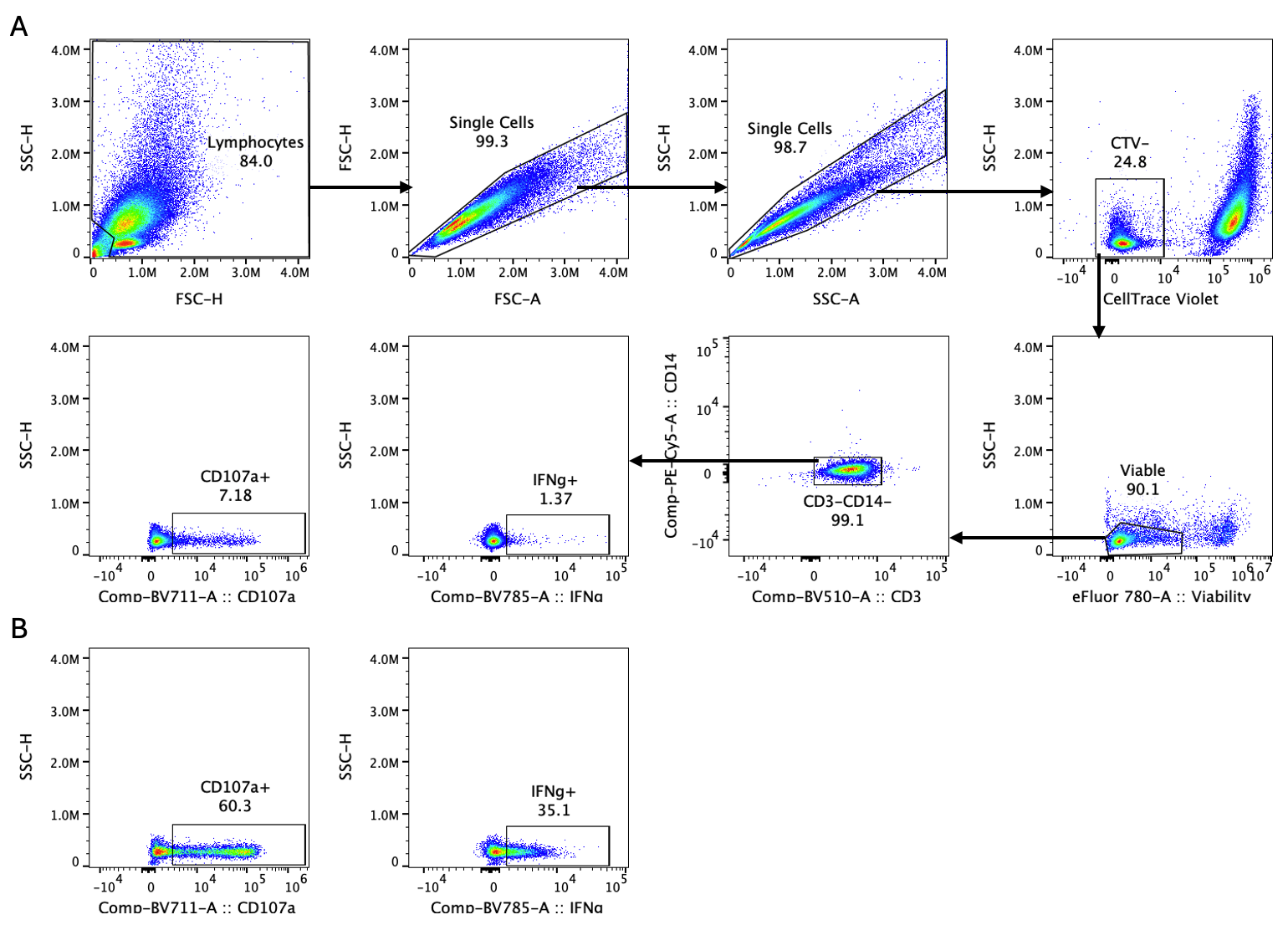


**Fig. S1. Assessing NK ADCC functional responses in COVID-19 infections.** (**A**) Representative flow cytometry plots demonstrating gating scheme for identifying CD107a and IFNγ responses in NK cells following treatment with Brefeldin A and monensin and co-culturing with uncoated, CellTrace Violet (CTV)-labeled Raji cells for 6 hours. (**B**) Representative flow cytometry plots depicting CD107a and IFNγ responses in NK cells following treatment with Brefeldin A and monensin and co-culturing with rituximab-coated, CTV-labeled Raji cells for 6 hours.


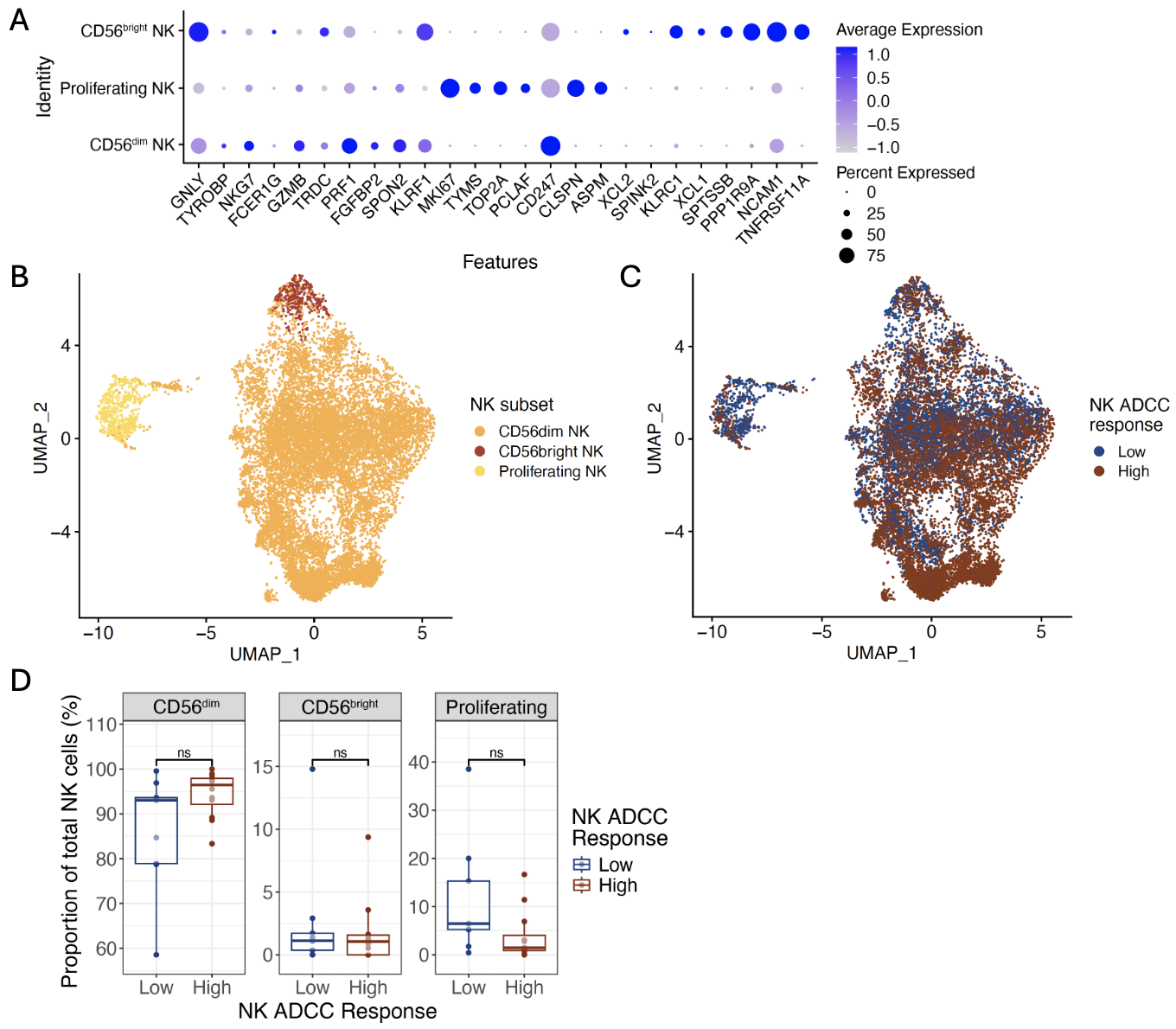


**Fig. S2. Evaluating NK subsets from participants with low versus high ADCC responses.** (**A**) Dotplot depicting gene features of NK subsets (annotations derived from PBMC reference mapping). (**B** and **C**) UMAP projection of NK cells in scRNA-seq dataset colored by NK subsets (**B**) and NK cell ADCC response, where low (n = 9) and high (n = 12) correspond to below and above mean ADCC degranulation responses, respectively (**C**). (**D**) Boxplots depicting the proportion of each NK cell subset out of total NK cells from participants with low (n = 9) or high (n = 12) NK cell ADCC responses. ns, not significant by Wilcoxon Rank-Sum test.

**
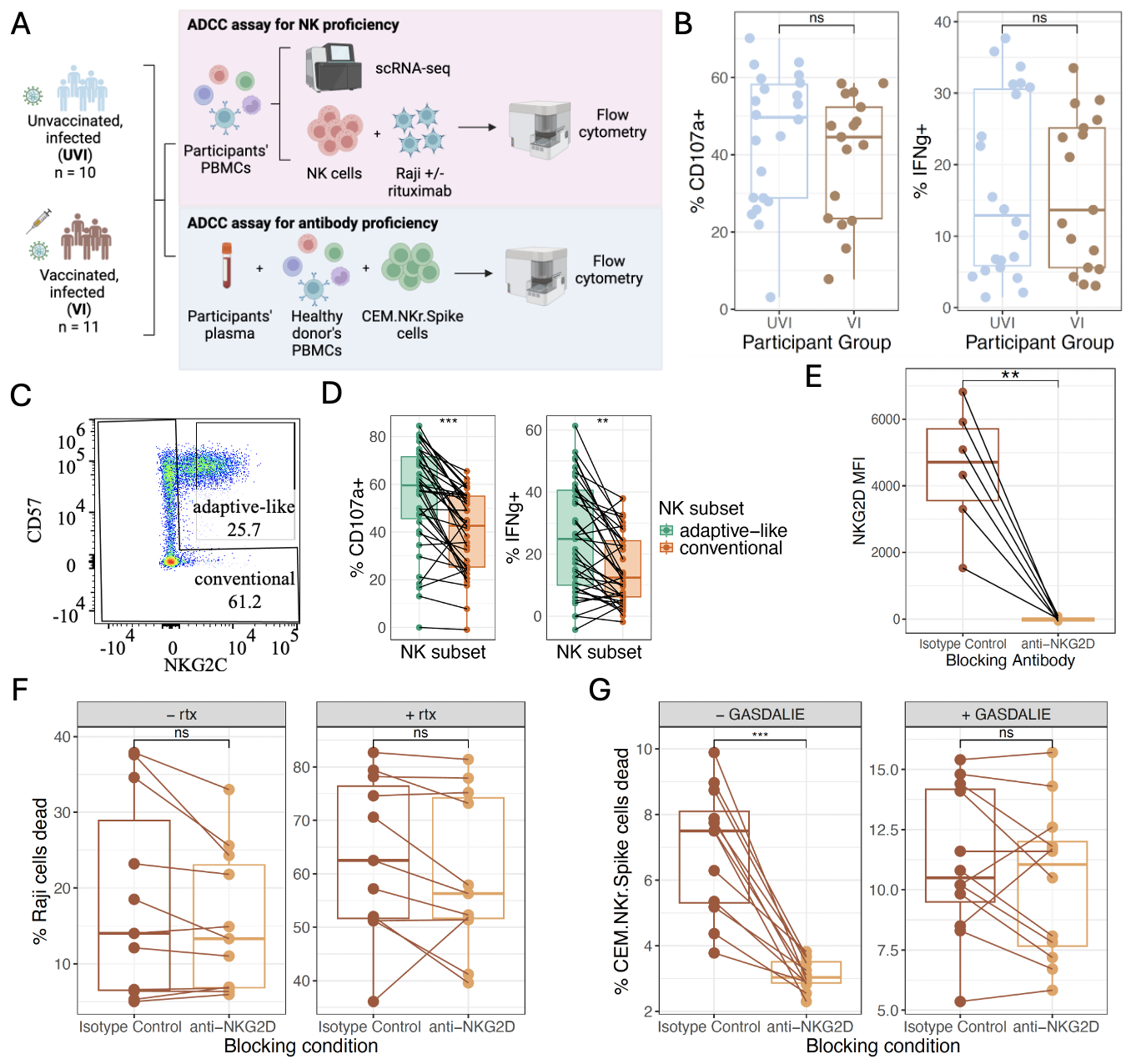
**

**Fig. S3. Adaptive-like NK cells and NKG2D function in ADCC responses.** **(A)** Pipeline of PBMC and plasma sample processing for paired scRNA-seq and ADCC functional assays, stratified by vaccination status. **(B)** Percent CD107a^+^ and IFNγ^+^ in participants’ NK cells measured during ADCC assay described in Fig. 1 in UVI (n = 10) and VI (n = 11) participant groups. **(C)** Representative flow cytometry plot demonstrating gating for adaptive-like and conventional NK cells. (**D**) Percent CD107a^+^ and IFNγ^+^ in participants’ adaptive-like and conventional NK cells (n = 21) measured during ADCC assay described in Fig. 1. **(E)** Boxplots depicting NKG2D median fluorescence intensity (MFI) in NK cells (n = 6) blocked with an isotype control or a monoclonal anti-NKG2D antibody. (**F** and **G**) Boxplots depicting percent dead CellTrace Violet-labeled Raji (n = 11) (**F**) and CEM.NKr.Spike (n = 12) (**G**) target cells when co-cultured with NK cells treated with isotype control or NKG2D blocking antibodies in the absence or presence of monoclonal ADCC-mediating antibodies for 3 hours and stained with a viability dye. ns, not significant; **, p <= 0.01; ***, p <= 0.001 by Wilcoxon Rank-Sum test.

**
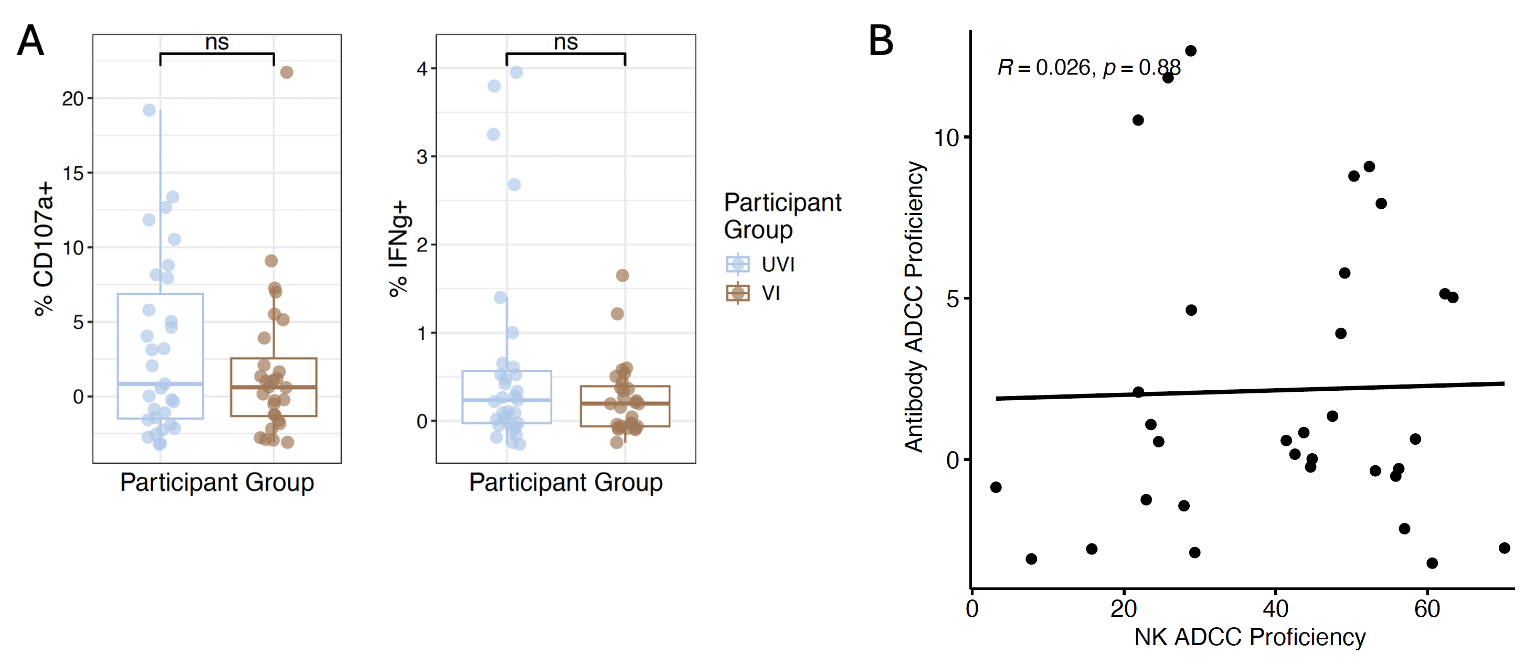
**

**Fig. S4. Determinants of participants’ plasma-mediated ADCC responses. (A)** Boxplots depicting the percent of healthy donor PBMCs staining positive for CD107a and IFNγ when co-cultured with CEM.NKr.Spike cells in the presence of participants’ plasma at 1:250 dilution at a 10:1 PBMC:CEM.NKr.Spike cell ratio for 4 hours, stratified by vaccination status. (**B**) Scatterplot depicting the correlation between participants’ NK cell versus antibody proficiency in mediating ADCC responses. ns, not significant by Wilcoxon Rank-Sum test.

**Table S1. Cohort demographics. WHO, World Health Organization; SD, standard deviation; BMI, body mass index.**

**
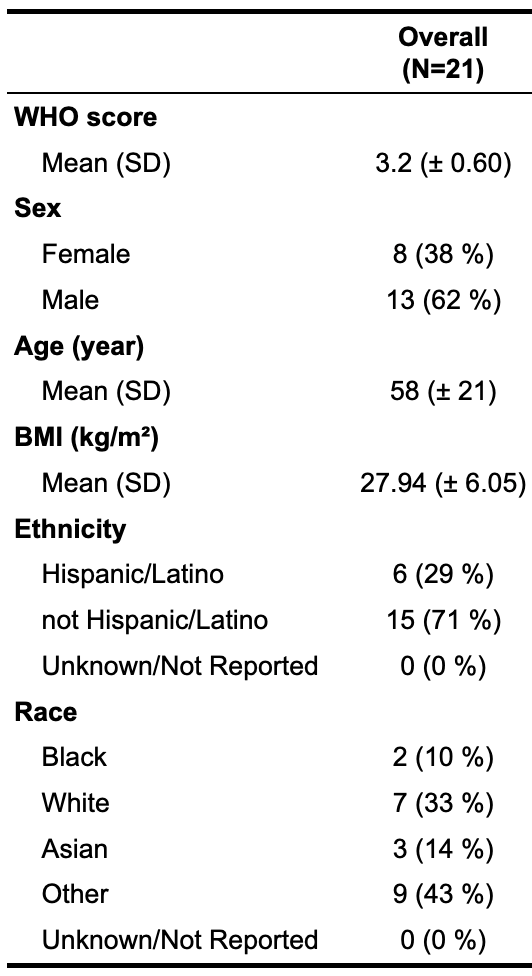
**

**Table S2. Flow cytometry antibody panel for NK cell ADCC functional assay.**

| **Antibody** | **Clone** | **Fluorophore** | **Volume (µL out of 100 µL)** | **Company** | **Catalog Number** |
| --- | --- | --- | --- | --- | --- |
| CD107a | H4A3 | Brilliant Violet 711 | 0.25 (out of 200 µL) | BioLegend | 328640 |
| CD3 | OKT3 | Brilliant Violet 510 | 0.25 | BioLegend | 317332 |
| CD56 | HCD56 | Brilliant Violet 605 | 0.125 | BioLegend | 362538 |
| CD16 | 3G8 | FITC | 0.5 | BioLegend | 302006 |
| CD14 | 61D3 | PE-Cyanine5 | 0.5 | Thermo Scientific | 15-0149-41 |
| CD19 | HIB19 | APC | 1 | Thermo Scientific | 17-0199-42 |
| NKG2C | 134591 | PE | 0.5 | R&D Systems |  |
| CD57 | HNK-1 | PE/Dazzle 594 | 0.125 | BioLegend | 359620 |
| IFNγ | 4S.B3 | Brilliant Violet 785 | 0.125 | BioLegend | 502542 |

**Table S3. Flow cytometry antibody panel for NK cell ADCC killing assay.**

| **Antibody** | **Clone** | **Fluorophore** | **Volume (µL out of 50 µL)** | **Company** | **Catalog Number** |
| --- | --- | --- | --- | --- | --- |
| CD3 | OKT3 | PE-Cyanine5 | 0.125 | BioLegend | 317356 |
| CD56 | HCD56 | Brilliant Violet 605 | 2 | BioLegend | 362538 |
| CD16 | 3G8 | Alexa Fluor 700 | 0.25 | BioLegend | 302026 |
| NKG2D | 1D11 | PE | 1 | BioLegend | 320806 |

**Table S4. Flow cytometry antibody panel for plasma ADCC assay.**

| **Antibody** | **Clone** | **Fluorophore** | **Volume (µL out of 50 µL)** | **Company** | **Catalog Number** |
| --- | --- | --- | --- | --- | --- |
| CD107a | H4A3 | Brilliant Violet 711 | 0.25 (out of 200 µL) | BioLegend | 328640 |
| CD3 | OKT3 | Brilliant Violet 510 | 0.25 | BioLegend | 317332 |
| CD56 | HCD56 | Brilliant Violet 605 | 0.125 | BioLegend | 362538 |
| CD16 | 3G8 | FITC | 1 | BioLegend | 302006 |
| CD14 | 61D3 | PE-Cyanine5 | 0.5 | Thermo Scientific | 15-0149-41 |
| CD19 | HIB19 | APC | 1 | Thermo Scientific | 17-0199-42 |
| IFNγ | 4S.B3 | Brilliant Violet 785 | 0.125 | BioLegend | 502542 |
